# Sulforaphane Inhibits Inflammatory Responses of Primary Human T-Cells by Increasing ROS and Depleting Glutathione

**DOI:** 10.3389/fimmu.2018.02584

**Published:** 2018-11-14

**Authors:** Jie Liang, Beate Jahraus, Emre Balta, Jacqueline D. Ziegler, Katrin Hübner, Norbert Blank, Beate Niesler, Guido H. Wabnitz, Yvonne Samstag

**Affiliations:** ^1^Section Molecular Immunology, Institute of Immunology, Heidelberg University, Heidelberg, Germany; ^2^Division of Rheumatology, Department of Internal Medicine V, Heidelberg University, Heidelberg, Germany; ^3^Department of Human Molecular Genetics, Heidelberg University, Heidelberg, Germany; ^4^nCounter Core Facility, Department of Human Molecular Genetics, Heidelberg University, Heidelberg, Germany

**Keywords:** sulforaphane, primary human T-cells, reactive oxygen species, glutathione, T_H_17, rheumatoid arthritis

## Abstract

The activity and function of T-cells are influenced by the intra- and extracellular redox milieu. Oxidative stress induces hypo responsiveness of untransformed T-cells. Vice versa increased glutathione (GSH) levels or decreased levels of reactive oxygen species (ROS) prime T-cell metabolism for inflammation, e.g., in rheumatoid arthritis. Therefore, balancing the T-cell redox milieu may represent a promising new option for therapeutic immune modulation. Here we show that sulforaphane (SFN), a compound derived from plants of the Brassicaceae family, e.g., broccoli, induces a pro-oxidative state in untransformed human T-cells of healthy donors or RA patients. This manifested as an increase of intracellular ROS and a marked decrease of GSH. Consistently, increased global cysteine sulfenylation was detected. Importantly, a major target for SFN-mediated protein oxidation was STAT3, a transcription factor involved in the regulation of T_H_17-related genes. Accordingly, SFN significantly inhibited the activation of untransformed human T-cells derived from healthy donors or RA patients, and downregulated the expression of the transcription factor RORγt, and the T_H_17-related cytokines IL-17A, IL-17F, and IL-22, which play a major role within the pathophysiology of many chronic inflammatory/autoimmune diseases. The inhibitory effects of SFN could be abolished by exogenously supplied GSH and by the GSH replenishing antioxidant N-acetylcysteine (NAC). Together, our study provides mechanistic insights into the mode of action of the natural substance SFN. It specifically exerts T_H_17 prone immunosuppressive effects on untransformed human T-cells by decreasing GSH and accumulation of ROS. Thus, SFN may offer novel clinical options for the treatment of T_H_17 related chronic inflammatory/autoimmune diseases such as rheumatoid arthritis.

## Introduction

Sulforaphane (SFN) is a natural compound obtained from cruciferous vegetables like broccoli, watercress, brussels sprouts, cabbage, and cauliflower. It has the ability to induce phase II anti-oxidant enzymes and exerts anti-proliferative effects on cancer cells *in vitro* (1–3). Reactive oxygen species (ROS) promote tumor development and progression, which was the rationale of the hypothesis that the ROS-detoxification process induced by SFN might be useful as an adjuvant during anti-cancer therapy. In several phase I and II clinical trials, the therapeutic benefit of SFN has been evaluated for healthy individuals and cancer patients (4, 5). However, a beneficial effect for cancer patients could not be documented in these studies. One possible explanation that has been discussed is a limited SFN concentration or pharmacokinetics in the patients (5). It is also known that the control of tumors is highly dependent on the immune system. Thus, if immune cells would be inhibited by SFN, this immunosuppression could outweigh the anti-tumor effects. However, effects of SFN on the immune system of cancer patients were not considered.

Recently, some studies have provided first hints that SFN is indeed able to modulate the immune system. Kumar et al. demonstrated that *in vitro* the development of human myeloid-derived suppressor cells (MDSCs) from CD14+ monocytes cultured in glioma conditioned medium was inhibited by SFN, which may enhance T-cell proliferation (6). On the other hand, the study by Pal et al. suggested that effects induced by SFN eventually shifted human monocyte polarization to a direction specific to M2 macrophages, promoting an anti-inflammatory phenotype (7). Geisel et al. reported that in a murine system, SFN led to diminished IL-12 and IL-23 expression by dendritic cells (DCs) eventually interfering with pro-inflammatory immune responses (8). Yet, a direct effect of SFN on mouse T-cells was not observed. In line with these latter findings, SFN was found to ameliorate murine experimental arthritis (9) and experimental autoimmune encephalomyelitis (EAE) (8, 10). In contrast to the murine system, SFN also seemed to have a direct inhibitory effect on synovial T-cells derived from rheumatoid arthritis (RA) patients (9). However, the effect of SFN on primary human T-cells from healthy donors was so far not investigated. Given enormous species differences, this aspect is critical for estimating its potential clinical effects.

The molecular principle of how SFN acts in different cell types is as yet only partly understood. Nuclear factor erythroid 2 (NFE2)-related factor 2 (NRF-2) was identified as one target of SFN in murine lymphocytes, murine DCs and cancer cells (11–13). NRF-2 is a leucine-zipper protein that is activated by oxidative stress and induces transcription of genes coding for anti oxidant proteins. Consistent with this, SFN treatment has been shown to boost the ROS-scavenger glutathione (GSH) in murine DCs, and also to result in high expression of the antioxidant protein heme oxygenase-1 (HO-1) (12). In contrast, another study using murine spleen lymphocytes demonstrated that 20 μM SFN rather increased the basal levels of intracellular ROS in murine spleen lymphocytes (11).

Taken together, the existing data create a confusing picture of the effects of SFN on the intracellular redox homeostasis, which might be due to the different systems used, i.e., murine vs. human cells, adaptive vs. innate immune cells or tumor cells vs. primary cells. However, an exact knowledge of the SFN effect on the redox-regulation in human T-cells is crucial to estimate its clinical relevance in T-cell related diseases, since the redox balance strongly modulates T-cell functions (14). In this regards, we have shown earlier that reducing conditions favor activation of primary human T-cells (15), whereas oxidative stress leads to hyporesponsiveness or even cell death of primary human T-cells (16, 17). In line with these findings, it has recently been postulated that low ROS levels in RA patient derived T-cells connects cellular metabolism with auto-aggressive T-cell immunity including biased differentiation of T-cells into IFN-γ and IL-17-producing inflammatory cells (18). Thus, the main purpose of our current study was to investigate whether SFN may serve as a novel means to influence the redox-balance and thereby the function of primary human T-cells.

Here, we show that SFN suppressed costimulation-induced activation and proliferation of primary human T-cells without having cytotoxic effects. In particular, expression of T_H_17-related proinflammatory genes were significantly diminished. These effects are attributable to redox regulation by SFN, since (i) SFN induced ROS and in parallel GSH depletion in primary human T-cells and (ii) thiol-containing antioxidants abolished the immunosuppressive effect of SFN. Taken together, our data imply that SFN may act as an immunosuppressive agent for primary human T-cells by regulating the T-cell redox equilibrium. This suppression of T-cell functions by SFN may be beneficial to control auto-inflammatory diseases such as RA.

## Materials and methods

### Reagents and antibodies

SFN was purchased from LKT Laboratories (St. Paul, MN). The following chemicals were obtained from Sigma-Aldrich: N-acetylcysteine (NAC), Tiron, Trolox, N-ethyl maleimide (NEM) and 4′,6-Diamidino-2-phenylindole (DAPI). GSH, 5-(and-6)-chloromethyl-2′, 7′-dichlorodihydro-fluorescein diacetate, acetyl ester (CM-H_2_DCFDA), ThiolTracker™ Violet and RPMI 1640 were purchased from Thermo Fisher Scientific. The cellular ROS/Superoxide detection assay kit was purchased from Abcam and carboxyfluorescein diacetate succinimidyl ester (CFSE) was from Invitrogen. SiR-actin was ordered from Cytoskeleton Inc. Fetal bovine serum (FBS) was bought from PAN-Biotech and BD FACS^TM^ lysing solution from BD Bioscience. IL-2 was purchased from Peprotech. Human CD8/NK and T_H_ cytokines panel were from BioLegend. Direct-zol™ RNA MiniPrep kit was purchased from QIAGEN and nCounter® GX Human Immunology v2 panel from nanoString Technologies. Vivaspin 6 (10,000 MWCO) columns were bought from Sartorius.

Antibodies employed in this study were specific for the following molecules: CD3 (clone OKT3 mouse mAb), CD28 (clone 28.2, BD Pharmingen), isotype control antibodies IgG1 and IgG2a (mouse mAb, BD Biosciences), cysteine sulfenic acid (rabbit polyclonal antibody, Merck), Prx1 (peroxiredoxin1, rabbit polyclonal antibody, Invitrogen), Trx (thioredoxin, Clone 2G11, mouse mAb, BD Bioscience), phospho-STAT3 (pSTAT3, #9131, Cell Signaling Technology), STAT3 (#9139, Cell Signaling Technology), and GAPDH (clone 6C5, mouse mAb, Invitrogen). For the secondary antibodies, donkey anti-rabbit IgG (H+L)-AF488 antibody was purchased from Dianova. IRDye® 800CW donkey anti-rabbit and IRDye® 680CW donkey anti-mouse were purchased from LICOR Biosciences. 7-AAD, Annexin-V and all fluorescently labeled antibodies were bought from BD Bioscience.

### Primary human T-cell preparation and cell culture

Human peripheral blood mononuclear cells (PBMCs) were obtained by Ficoll–Hypaque (Linaris, Wertheim-Bettingen, Germany) density-gradient centrifugation of heparinized blood from healthy volunteers. T-cells were isolated using the pan T-cell isolation kit purchased from Miltenyi Biotec (Bergisch Gladbach, Germany) as per the manufacturer's instructions. The human T-leukemia cell line Jurkat ACC282 and B-leukemia cell line Raji were grown in RPMI 1640 complete medium containing 10% FBS at 37°C and 5% CO_2_. This study was approved by the Ethics Committee of the Heidelberg University (S-269/2015).

### Sampling of blood from RA patients

Heparinized peripheral blood was collected under aseptic conditions from patients with RA. Informed consent for use of the cells was obtained from all RA patients included in this study. This study was approved by the Ethics Committee of the Heidelberg University (S-119/2017).

### T-cell costimulation

To co stimulate human peripheral T-cells, microplates (Nunc, Wiesbaden, Germany) were pre-coated with goat anti-mouse IgG+M antibody followed by blocking with complete RPMI medium (RPMI+10% FBS), coating with anti-CD3 (20 ng/mL), and anti-CD28 (5 μg/mL) antibodies or the respective isotype controls. T-cells were spun down on the antibodies and incubated at 37°C for the indicated time points.

### Cell viability assay

Primary human T-cells or Jurkat T-leukemia cells were cultured in 200 μl complete RPMI medium for the indicated time points with or without SFN (5% CO_2_ and 37°C). For viability assays, cells were washed with pre-warmed phosphate-buffered saline (PBS), resuspended in Annexin-V binding buffer containing 7-AAD and Annexin-V and incubated 15 min at room temperature (RT). After washing once with Annexin-V binding buffer, cells were acquired via flow cytometry (LSRII, BD Bioscience, Heidelberg, Germany) and data were analyzed with FlowJo X (FlowJo LLC, Ashland, OR, USA).

### T-cell/APC conjugate formation

Conjugates were formed between T-cells and staphylococcus aureus enterotoxin B (SEB) loaded Raji cells as described previously (19). Briefly, T-cells were pre-incubated in the absence or presence of 10 μM SFN for 1 h and Raji cells were loaded with 5 μg/ml SEB or kept unloaded. Then, T-cells and Raji cells were coincubated at a ratio of 1:1 for 45 min at 37°C. Cells were fixed using 1.5% PFA and stained with anti-CD3-APC and anti-CD19-PerCP-Cy5.5 antibodies. Cell couple formation was determined by flow cytometry. Double positive events (CD3^+^CD19^+^) were counted as cell couples.

### Analysis of immune synapses (IS) by multispectral imaging flow cytometry (MIFC)

Conjugates were formed between T-cells and SEB loaded Raji cells as described above. Briefly, T-cells were incubated in the absence or presence of 10 μM SFN for 1 or 24 h at 37°C. Then 1 × 10^6^ T-cells/sample were mixed at a 1:1 ratio with Raji cells that were either loaded with 5 μg/ml SEB or kept unloaded, and the cells mixture was incubated for 45 min at 37°C to allow for IS formation. Then cells were fixed in 1.5% PFA, and stained with anti-CD3-PE-TxR, anti-LFA-1-FITC, and DAPI. Thereafter, cells were subjected to MIFC (IS100, Amnis Corp., Seattle, WA, USA) and as many as 15,000 images per sample were acquired. Subcellular localization of proteins was analyzed with IDEAS 6.0 software (Amnis, Seattle, WA, USA) as described previously (20).

### Detection of T-cell activation

T-cells were kept untreated or treated with SFN and co-stimulated with plate bound anti-CD3/CD28 antibodies (for details see above). Activation of T-cells was evaluated by the expression of CD25 and CD69. Briefly, T-cells were washed once with FACS wash (FW) buffer (0.5% BSA, 0.5% FBS, and 0.07% NaN_3_ in 1 X PBS) to remove the culture medium. The cell pellet was resuspended in FW containing anti-CD25-APC and anti-CD69-PE antibodies and incubated for 20 min at RT. Thereafter, cells were washed and analyzed by flow cytometry. To determine the total amount of CD25 and CD69 (cell surface plus intracellular), cells were fixed with 1.5% PFA and permeabilized with FWS (FW containing 0.1% saponin) prior to antibody staining.

### Assessment of T-cell proliferation

T-cells were washed with pre-warmed PBS, resuspended in PBS, loaded with 1 μM CFSE and incubated for 15 min at 37°C. After washing once with PBS to remove unbound CFSE, SFN was added for 30 min and the cells were seeded on 96-well microplates coated with anti-CD3/CD28 antibodies for co-stimulation as described above. Proliferation was determined after 3 days of incubation using flow cytometry. The proliferation index was calculated according to the instruction of FlowJo documentation. Briefly, the number of cells at the beginning of cell culture (Ns = G0 + G1/2 + G2/4 + G3/8), the total number of divisions (Nt = (G1/2)^*^1 + (G2/4)^*^2 + (G3/8)^*^3) and the number of cells that subjected to division (Nd = Ns–G0) were calculated first. The proliferation index results from the ratio of Nt and Nd.

### Cytokine assay

Human CD8/NK and T_H_ cytokine panels were used to detect secreted cytokines. To this end, SFN treated T-cells or untreated T-cells (1 × 10^5^ T-cells/100 μl), concentrations as indicated, were co-stimulated with crosslinked anti-CD3/CD28 antibodies. After 2 days of incubation, the samples were centrifuged, cell-free supernatants were collected and immediately aliquoted and stored at −80°C. The cytokine assay was performed using undiluted samples and 96-well U-bottom microplates as per the manufacturer's instructions.

### Detection of intracellular ROS levels

Intracellular ROS levels were detected by using the ROS detection reagent CM-H_2_DCFDA. T-cells (1 × 10^6^ T-cells/ml) or Jurkat T-leukemia cells were washed with PBS and the cell pellet was re-suspended in PBS. CM-H_2_DCFDA was added to a final concentration of 5 μM, and the cell suspension was incubated for 15 min at 37°C in the dark. After washing twice with PBS, the cell pellet was re-suspended in RPMI 1640 complete medium and SFN was added as indicated in the respective figures. As control, we used H2O2 (50 μM for PBTs and 50 μM to 200 μM for Jurkat T-leukemia cells). The fluorescence intensity was immediately determined by flow cytometry and analyzed with FlowJo X.

For detecting of the intracellular ROS level of lymphocytes in whole blood, heparinized peripheral blood from RA patients was pre-treated with 10 μM SFN or left untreated for 5 min (Fresh blood was used up to 2 h after blood donation) at RT. Alexa Fluor® 700-CD45 antibody was used to stain leukocytes, and CM-H_2_DCFDA was used to stain intracellular ROS. After staining for 15 min, blood samples were proceeded to erythrocyte lysis with FACS^TM^ lysing solution, washed twice with FW, and measured by flow cytometry and analyzed with FlowJo X.

### Detection of intracellular GSH levels

The intracellular GSH levels were detected using ThiolTracker^TM^ violet dye according to manufacturer's instructions. Briefly, T-cells or Jurkat T-leukemia cells were kept untreated or treated with the indicated concentration of SFN in RPMI 1640 complete medium. After SFN treatment for the indicated time points, cells were spun down and the supernatant was removed. Cells were rinsed twice with pre-warmed PBS and re-suspended in 100 μl PBS containing 2 μM ThiolTracker^TM^ violet dye and incubated for 15 min at 37°C. After washing and resuspending in 100 μl PBS, cells were immediately measured using flow cytometry and analyzed with FlowJo X.

### Assessment of cysteine sulfenylation

For assessment of global sulfenylation on cysteine thiols, a dimedone specific antibody was used. Quantification of dimedone signal was performed using flow cytometry and super-resolution microscopy (Structured illumination microscopy, SIM using N-SIM, Nikon). For flow cytometric analysis, 2 × 10^5^ T-cells were treated with 10 μM SFN or kept untreated for 10 min at 37°C. Thereafter, samples were washed and fixed for 10 min with 1.5% PFA containing 0.25% DMSO and 5 mM dimedone, and permeabilized with FWS. Rabbit anti-dimedone serum (1:1,500) and anti-rabbit AF488 (1:1,200) were used to detect the dimedone signal, and data were analyzed with FlowJo X. For microscopic analysis, 2 × 10^5^ T-cells were initially allowed to adhere on coated coverslips and immunocytochemistry protocols were performed as described (21). After adherence on the coverslips, SFN treatment, fixation, and permeabilization were performed as described above. Thereafter, the samples were stained with rabbit anti-dimedone serum (1:1,500) and anti-rabbit AF488 (1:1,200) sequentially. During the process of secondary antibody staining, nuclei were stained with DAPI (1:5,000), and F-actin was detected by incubation with SiR-actin (500 nM). Images of the cells were acquired using an N-SIM microscope equipped with a 100x objective (NA 1.49).

### Kinetic trapping by Trx1

The Trx1 trapping mutant (Trx1 C35S) was purified and loaded on streptavidin beads as described (22). Briefly, T-cells from healthy donors were treated with the indicated concentrations of SFN or H_2_O_2_ for 10 min at RT. Thiols within the cells were alkylated using 100 mM NEM for 5 min. Excess amount of NEM was removed by extensive washing with PBS. Thereafter, the cells were lysed in TBS with 1% Triton X-100 and protease inhibitor cocktail for 30 min on ice. The cytoplasmic fraction was collected by centrifugation at 10,000 × g for 10 min. The postnuclear lysates were loaded on streptavidin beads that had been preloaded with Trx1 trapping mutant (Trx1 C35S SBP 6x His). The Trx1 loaded beads and lysates were incubated for 3 h on a rotator at 4°C, then the reaction was stopped by adding NEM to a final concentration of 20 mM and incubating on ice for 5 min. Unbound proteins were washed out extensively with the following buffers stepwise: 1% Triton X-100, 500 mM NaCI, 1 mM NEM, 1 M Urea in 1x TBS, 1% Triton X-100, 1 mM NEM in 1x TBS, and 1% Triton X-100 in 1x TBS. The Trx1 C35S and kinetically trapped proteins were released from the streptavidin beads using excess amount of biotin in the elution step. Next, the eluted proteins were concentrated using protein concentrator vivaspin 6 (10,000 MWCO) columns. Finally, the samples were divided into two and mixed with 1x reducing (DTT) or nonreducing (without DTT) sample buffer. The preparates were loaded on SDS-PAGE and immunoblotted for the indicated proteins.

### Gene expression profiling

The nCounter® Nanostring GX Human Immunology v2 panel designed for the expression analysis of 579 immune and inflammation associated target genes and 15 internal reference control genes, was used for expression profiling. In brief, total RNA was extracted from all samples (1 × 10^6^ T-cells/sample) by Trizol per the manufacturer's instructions. Subsequently, nCounter® Nanostring based gene expression profiling was performed on 25 ng total RNA from each sample. All RNA samples were quantified by using Qubit™ RNA assay kits and quality control was performed on the Agilent 2100 Bioanalyzer system. Qualified samples were subjected to overnight hybridization reaction at 65°C, where 5 μl of total RNA samples were combined with 3 μl of nCounter® reporter CodeSet in 5 μl of hybridization buffer and 2 μl of nCounter® capture ProbeSet for a total reaction volume of 15 μl and incubated for 20 h. Ramp reactions down to 4°C. Afterwards, samples were purified and immobilized on a cartridge and data assessed on the nCounter® *SPRINT* Profiler. During sample processing, the instrument performs a number of tasks including liquid transfers, magnetic bead separation, and immobilization of molecular labels on the sample surface. This is followed by data collection with an automated fluorescence microscope and digital analysis system. The results then are exported as a comma separated values (CSV) file and analyzed using NanoString's analysis software nSolver 4.0 and R statistical software.

### Western blotting

For total lysate preparation, T-cells (1 × 10^6^ T-cells/ml) were washed with PBS and lysed using PBS with 1x reducing or non-reducing sample buffer. Then the total lysates were run on polyacrylamide gels, proteins were blotted on PVDF-membranes and the membranes were blocked in blocking buffer for 1 h. Afterwards, membranes were stained with primary antibodies against pSTAT3 (1:1,000), STAT3 (1:1,000), GAPDH (1:10,000), Prx1 (1:1,000), Trx1 (1:1,000), and respective secondary antibodies. The membranes were scanned by a Licor infrared scanner (LI-COR Biosciences).

### Statistical analysis

The statistical analysis was performed with GraphPad Prism version 6.00 (STATCON, Witzenhausen, Germany). Two groups were compared using *t*-test or paired *t*-test for matched observations. Multiple groups were compared using ANOVA. Heat maps were generated with R statistical software.

## Results

### SFN is not toxic to untransformed human T-cells but to jurkat T-leukemia cells

To elucidate the influence of SFN on untransformed human T-cells, we prepared freshly isolated peripheral blood T-cells and incubated these cells with SFN. In previous studies, concentrations of SFN up to 80 μM were used for different *in vitro* experiments. However, since a maximum concentration of 2.5 μM SFN was detected in human plasma after broccoli sprout consumption (23), we did not exceed 20 μM SFN in our current study.

**Figure 1:**
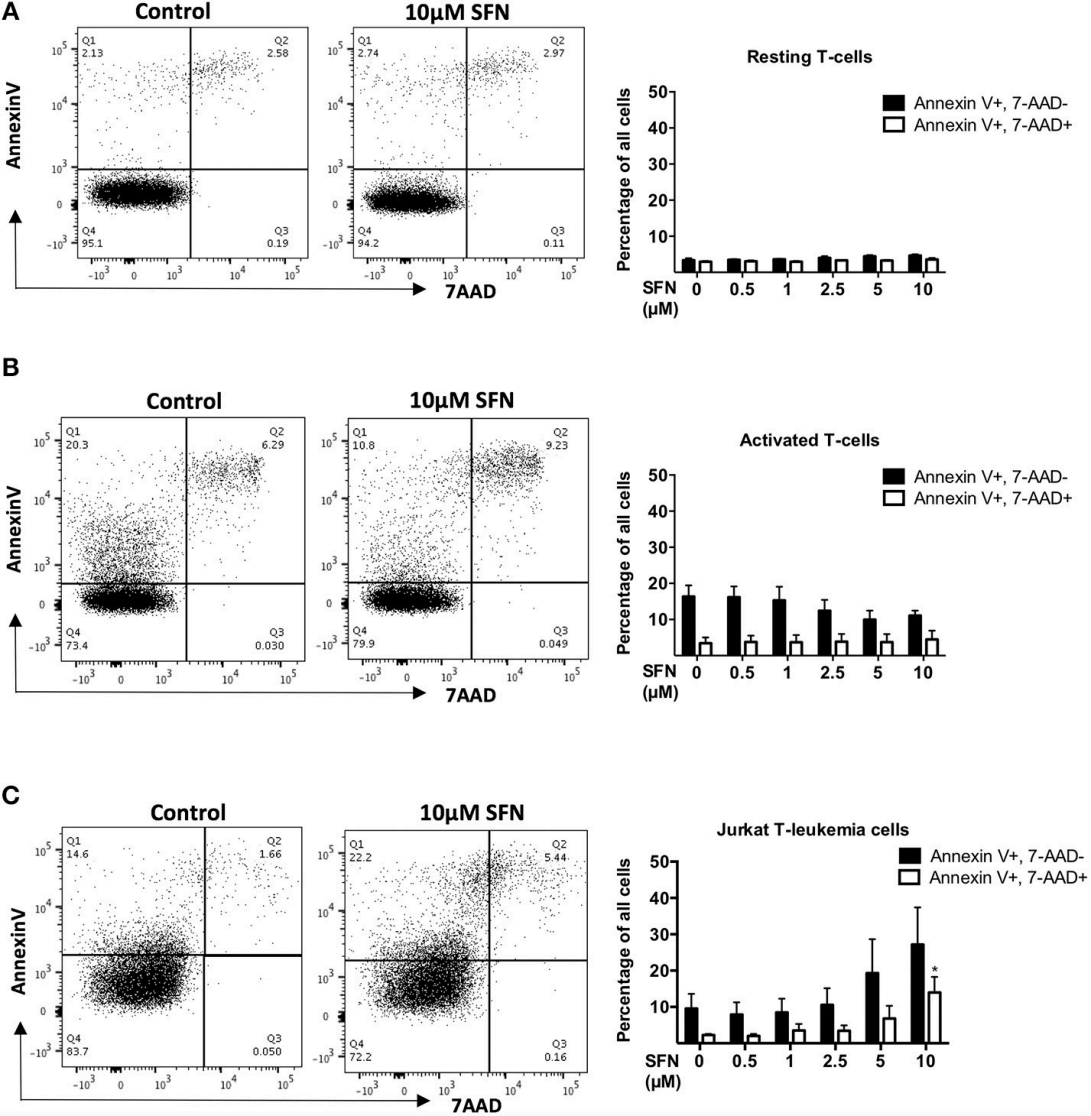
SFN is not cytotoxic to untransformed human T-cells but to Jurkat T-leukemia cells. **(A,B)** Flow cytometry analysis of T-cell viability, resting T-cells were treated with SFN as indicated and **(A)** kept unstimulated (resting T-cells) or **(B)** co-stimulated with anti-CD3(20 ng/ml)/CD28 (5 μg/ml) antibodies for 1 day (activated T-cells), thereafter stained with 7-AAD and Annexin-V. **(C)** Flow cytometry analysis of Jurkat T-leukemia cell viability. Cells were treated with SFN as indicated for 1 day, thereafter stained with 7-AAD and Annexin-V. Shown are representative flow cytometry dot plots (left panel) from three experiments and the corresponding quantification (right panel; *n* = 3; mean; SE; ^*^p < 0.05).

To investigate whether SFN is toxic to primary human T-cells, we first assessed T-cell viability after 1 day of SFN treatment. To this end, we used Annexin-V to specifically identify apoptotic cells, and 7-AAD to evaluate cells with progressive loss of membrane permeability, i.e., dead cells. Dual staining with 7-AAD and Annexin-V-PE showed that SFN had no cytotoxic effect on primary human T-cells up to the maximal concentration of 10 μM (Figures 1A,B). To substantiate our finding, we also measured the mitochondrial membrane potential of SFN treated T-cells using TMRM (Supplementary Figure 1). In line with the former assay, the mitochondrial membrane potential was not disturbed by SFN. Since cytotoxic effects of SFN were described for tumor cells such as breast cancer cells and prostate cancer cells (24, 25), and especially also for acute lymphoblastic leukemia cells (3), we also measured the effects of SFN on the viability of the Jurkat T-leukemia cell in parallel. Notably, although primary human T-cells did not show any increased levels of apoptosis (Figures 1A,B), there was a concentration-dependent increase in toxicity toward Jurkat T-leukemia cells (Figure 1C). This clearly shows that in physiologically relevant concentrations SFN negatively influences the survival of malignant T-cells but not of untransformed primary human T-cells.

### SFN does not impair T-cell/APC conjugate and immune synapse formation

**Figure 2:**
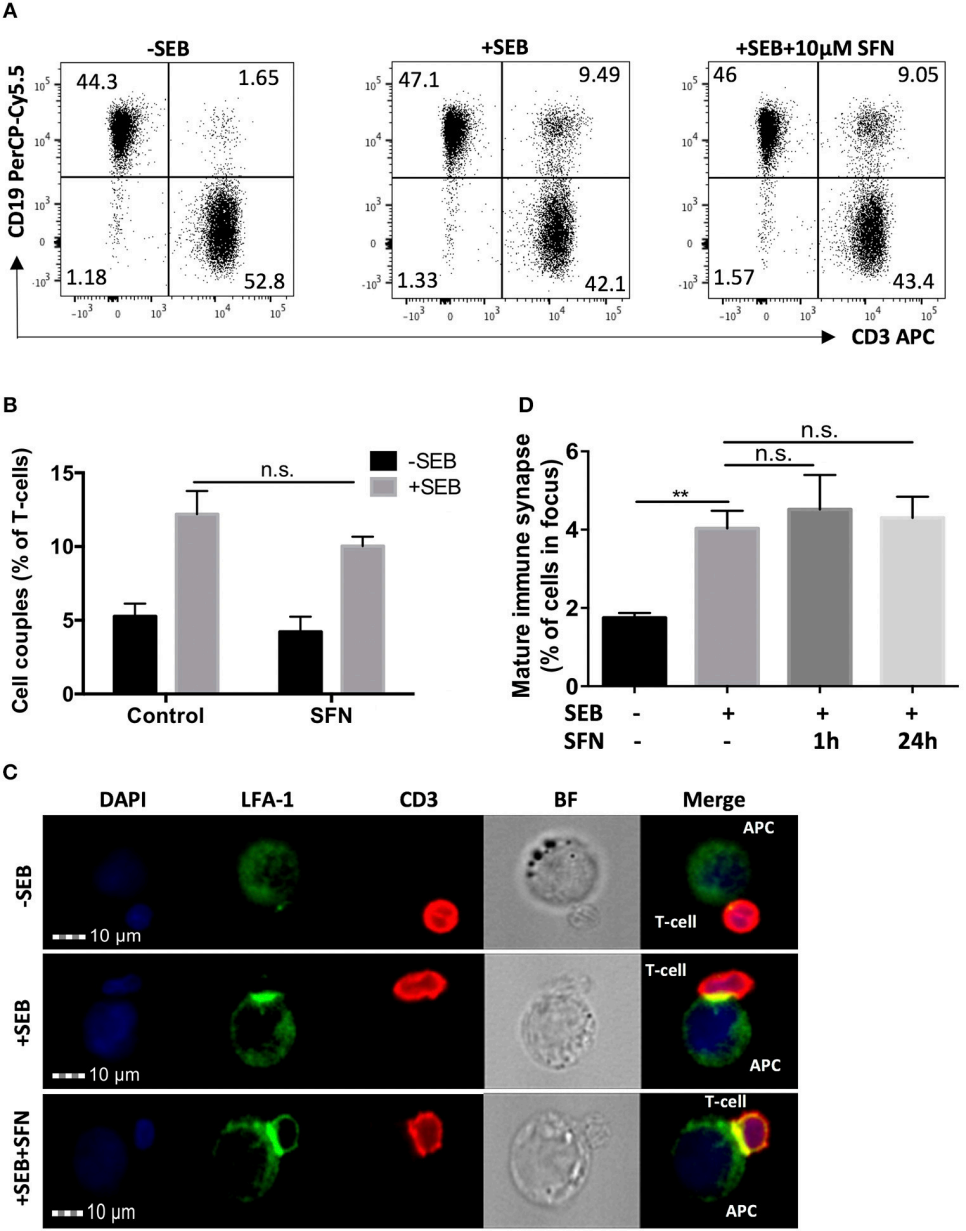
Intact immune synapse formation of SFN treated T-cells. **(A)** Dot plots of T-cell/APC couple formation were identified by flow cytometry-based conjugate assay via CD3 and CD19 expression. T-cells were treated without (left and middle panel) or with (right panel) 10 μM SFN prior to incubation with APCs in the absence (left panel) or presence (middle and right panel) of SEB. Dot plots are representative for three experiments. **(B)** Quantification of the couple formation between T-cells and APCs (*n* = 3; mean; SE). **(C)** Multispectral imaging flow cytometry analysis of mature synapse formation. T-cells were treated without (upper and middle panels) or with (lower panel) 10 μM SFN prior to incubation with APCs in absence (upper panel) or presence (middle and lower panels) of SEB. Cells were stained for nuclei (DAPI, blue), LFA-1 (green) and CD3 (red). The merged image represents the digital overlay of all three colors. Bright field (BF) and fluorescence images are representative for three experiments. **(D)** Quantification of the mature immune synapse between T-cells and APCs. At least 15,000 cells were acquired by imaging flow cytometry for each condition (*n* = 3; mean; SE; ^**^p < 0.01).

To evaluate the effects of SFN on T-cell activation, we analyzed early and late activation events. Crucial initial steps in that regard are the T-cell/APC (antigen-presenting cell) conjugate formation and maturation of an immune synapse (IS). To investigate this, T-cells were pre-treated with 10 μM SFN or kept untreated for 1 h, then incubated with SEB loaded Raji cells that served as APCs. T-cell/APC couples were identified by a flow cytometry-based conjugate assay (Figure 2A). Single CD3 or CD19 positive events represented solitaire T-cells or solitaire Raji cells, respectively, whereas CD3/CD19 double positive events arose from T-cell/APC conjugates. As expected, in the absence of SEB, only 1.65% T-cell/APC conjugates were found (Figure 2A, left part). In the presence of SEB, both the untreated and SFN treated group showed a clear formation of T-cell/APC conjugates, i.e., 9.49 or 9.05%, respectively (Figure 2A, middle and right part). To quantify the percentage of T-cell/APC conjugates, we acquired up to 10,000 T-cells in three independent experiments and found that T-cell/APC conjugate formation was not significantly influenced by SFN treatment (Figure 2B).

Although T-cell/APC conjugate formation can be measured using flow cytometry, this technique is limited for evaluating spatial informations which are important for analysing the formation of the immune synapse. We, therefore, took advantage of MIFC, which combines fluorescence microscopy and flow cytometry and is a suitable means for the analysis of immune synapse formation (26). By defining regions of interest, MIFC allows the spatial quantification of fluorescence signals within T-cells, and thus of the accumulation of receptors at the T-cell/APC interface. In brief, T-cell/APC couples were identified according to DAPI staining and CD3 expression. Then, the accumulation of the TCR/CD3 complex and LFA-1 in the T-cell/APC contact zone was used as measure for the formation of a mature immune synapse. As expected, while most T-cells showed no enrichment of TCR/CD3 and LFA-1 in the contact zone without SEB treatment, a clear receptor enrichment—and thus immune synapse maturation—was observed in the presence of SEB (Figures 2C,D). Consistent with the contact formation analysis by flow cytometry, SFN treatment did not impair the immune synapse formation.

### SFN inhibits CD25/CD69 expression and proliferation of primary human T-cells

**Figure 3:**
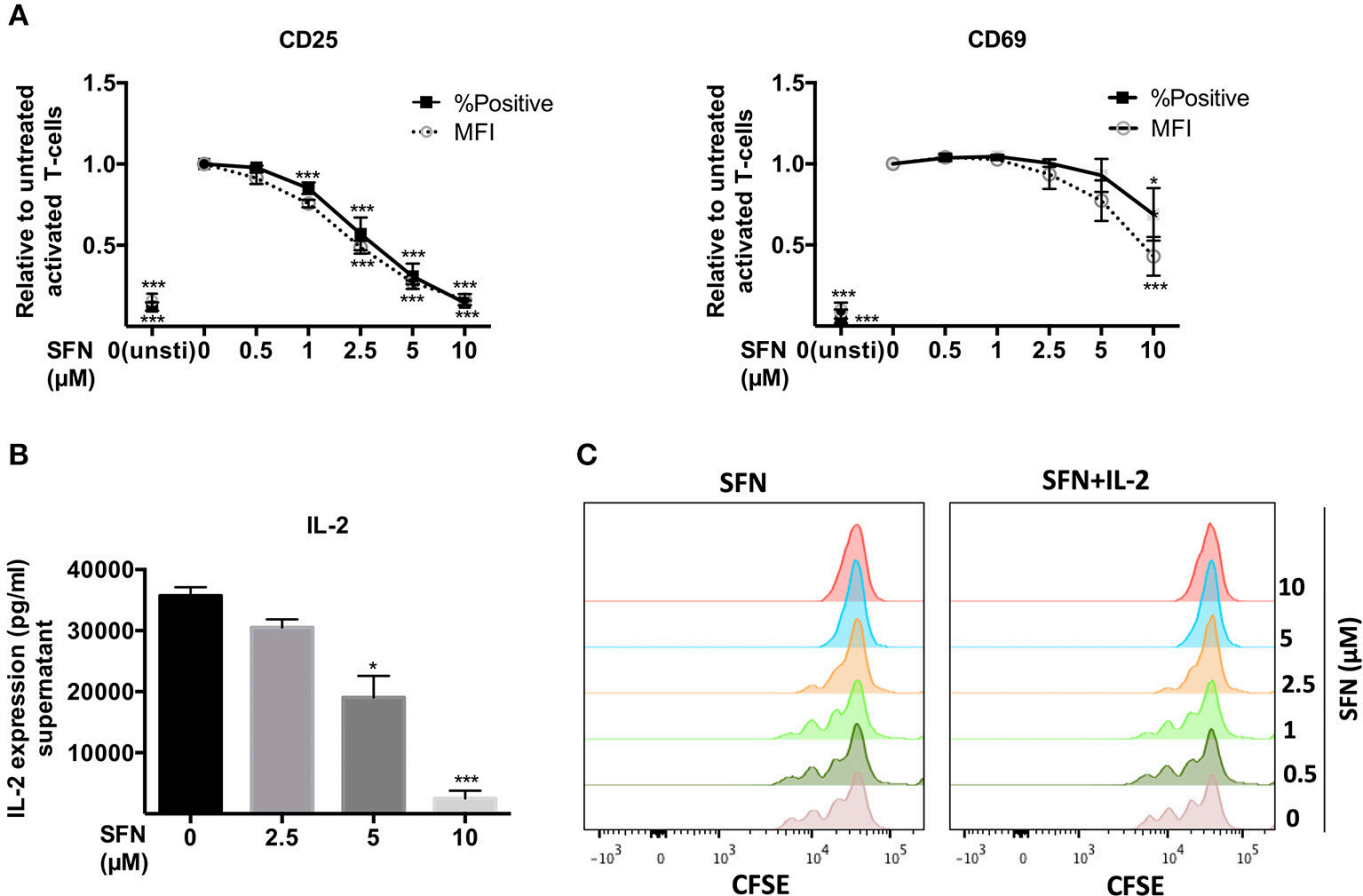
SFN inhibits activation of untransformed human T-cells. **(A)** Flow cytometry analysis of CD25 and CD69 surface expression in T-cells post 12 h stimulation with anti-CD3(20 ng/ml)/CD28(5 μg/ml) antibodies or unstimulated T-cells (unsti). Given are expression levels relative to the stimulated control (no SFN treatment) in terms of %positive cells (black square, solid line) and mean fluorescence intensities (MFI, open circle, dashed line) (*n* = 3; mean; SE; ^*^p < 0.05, ^***^p < 0.001). **(B)** Quantification of IL-2 production in the supernatant of T-cells without (black histogram) or with SFN (gray histograms) treatment as indicated (*n* = 3; mean; SE; ^*^p < 0.05, ^***^p < 0.001). **(C)** T-cell proliferation detected via staining of CFSE. T-cells were co-stimulated in the absence/presence of SFN without /with addition of exogenous IL-2. CFSE signals were measured by flow cytometry after 3 days of co-stimulation. Histograms are representative for three experiments.

Early T-cell activation events such as T-cell/APC conjugate and immune synapse formation were seemingly not changed by SFN. We, therefore, continued to investigate the T-cell activation process at later stages. To this end, we first analyzed the surface T-cell activation markers, CD25 and CD69. After 12 h of T-cell costimulation with anti-CD3/CD28 antibodies in the absence of SFN, CD25 and CD69 were strongly expressed, while their expression was diminished in a dose-dependent manner in the presence of SFN (Figure 3A). Notably, even 2.5 μM SFN, a concentration that can be found in human serum after broccoli sprout consumption, was sufficient to dampen the expression of CD25 (and slightly CD69) on the surface of T-cells. We have previously shown that not only the expression, but also the transport of CD25 and CD69 to the T-cell surface requires costimulation (19). To clarify whether the decreased level of CD25 and CD69 was due to impaired functioning of the surface transport or overall expression, we stained the total amount of these proteins within the cells, i.e., after permeabilization with FWS. The results were similar compared to the cell surface staining (Supplementary Figure 2), which indicates that SFN inhibited the expression of both proteins, CD25 and CD69. Next, we quantified the expression of the T-cell growth factor IL-2 in the supernatants of T-cells that were treated with various concentrations of SFN and then costimulated for 2 days. Figure 3B shows that the level of IL-2 in the supernatant of T-cells significantly decreased with increasing SFN concentrations.

T-cell activation initiates intracellular signaling cascades that ultimately result in T-cell proliferation. Labeling of T-cells with CFSE allows to monitor T-cell division over time. Thus, we pre-treated CFSE-labeled T-cells with SFN and detected the proliferation after 3 days of costimulation using flow cytometry. In line with the finding that expression of IL-2 and the IL-2 receptor CD25 were dampened by SFN, treatment with SFN significantly diminished T-cell proliferation compared to the control cells (Figure 3C, left panel). Again, even low SFN concentrations (2.5 μM) were sufficient to hamper T-cell proliferation. Notably, this phenotype could not be rescued by adding exogenous IL-2 (40 U/ml) to costimulated T-cells (Figure 3C, right panel). Moreover, the SFN-mediated decrease in CD25 and CD69 expression was not restored by addition of IL-2 (Supplementary Figure 3). Collectively, these results show that SFN interfered with T-cell activation events.

### SFN induces a pro-oxidative milieu in untransformed human T-cells

**Figure 4:**
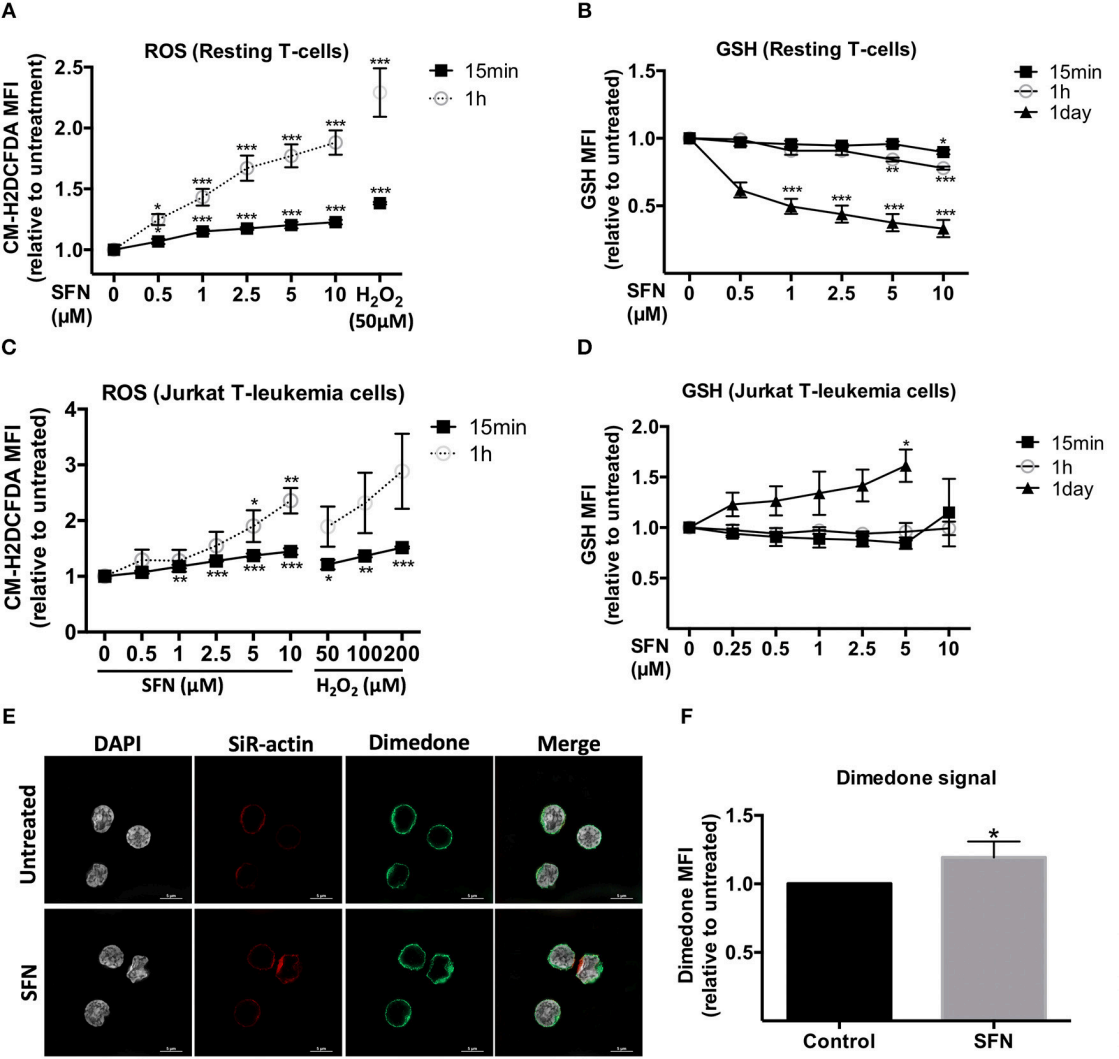
Influence of SFN on the redox state of untransformed human T-cells and Jurkat T- leukemia cells. **(A,C)** Intracellular ROS levels in T-cells and Jurkat T- leukemia cells, respectively, were assessed by flow cytometry. Cells were pre-loaded with CM-H_2_DCFDA and, thereafter, treated without or with SFN as indicated for different time points (*n* = 3; mean; SE; ^*^p < 0.05, ^**^p < 0.01, ^***^p < 0.001). **(B,D)** Intracellular GSH levels of T-cells and Jurkat T-leukemia cells, respectively, were assessed by flow cytometry. Cells were treated with SFN as indicated for different time points and stained with ThiolTracker^TM^ violet dye for 15 min (*n* = 3; mean; SE; ^*^p < 0.05, ^**^p < 0.01, ^***^p < 0.001). **(E)** Sulfenylated cysteines in T-cells were detected by microscopy. SFN treated T-cells were fixed and permeabilized, stained with DAPI (white), a dimedone specific antibody (green), and SiR-actin (blue). The merged image represents the digital overlay of all four colors. Images are representative for three experiments. **(F)** Quantititive analysis of cysteine sulfenylation by flow cytometry. Histogram shows the MFI relative to control of three independent experiments (*n* = 3; mean; SE; ^*^p < 0.05).

ROS are ubiquitously generated and recognized as important signaling molecules for T-cell activation, but excessive ROS generation or prolonged exposure to high ROS concentrations impair T-cell functions (27). SFN was previously reported to have mostly antioxidative effects by promoting Nrf-2 activation (13). Since the effect of SFN on the redox system of primary human T-cells remained unclear, we sought to evaluate the intracellular ROS level in untransformed human T-cells after SFN treatment. To this end, T-cells were loaded with the ROS-sensitive probe CM-H_2_DCFDA and treated with SFN prior to flow cytometric analysis. SFN treatment induced a significant accumulation of ROS in untransformed human T-cells in a concentration dependent manner (Figure 4A). At a concentration of 10 μM SFN, the intracellular ROS levels were comparable to those observed after treatment of 50 μM H_2_O_2_. The increased intracellular ROS levels after SFN treatment were also confirmed by another assay (cellular ROS/Superoxide detection kit, Abcam, data not shown).

We next examined the intracellular levels of the important ROS-scavenger GSH in T-cells using ThiolTracker^TM^ violet dye and flow cytometry. These experiments showed that SFN decreased the intracellular GSH levels in SFN treated T-cells compared to untreated T-cells (Figure 4B). Notably, whereas higher SFN doses were needed to diminish the GSH levels after 1 h, very low SFN concentrations (e.g., 1 μM) were sufficient to significantly interfere with the amount of GSH in T-cells after 1 day. Taken together, these data suggest that SFN has a pro-oxidative effect on untransformed human T-cells leading to an increase of ROS levels and depletion of the ROS-scavenger GSH. Interestingly, in Jurkat T-leukemia cells, SFN also increased the ROS levels (Figure 4C). However, in contrast to untransformed human T-cells, in which SFN treatment led to a decrease of GSH, the GSH level in Jurkat T-leukemia cells showed a significant increase after 1 day of SFN treatment (Figure 4D). This difference was also confirmed by another GSH specific reagent, namely thiol green dye (Supplementary Figure 4). This underpins our previous observation regarding cell viability (Figure 1) that SFN affects tumor cells and primary cells differently.

To further elucidate the pro-oxidative effect of SFN on untransformed human T-cells, we next investigated whether SFN promotes oxidation of cellular proteins. The first state of oxidation of thiols (sulfenylation) is reversible and transient. Sulfenylated cysteines are protected from further oxidation either by glutathionylation (S-glutathionylation) or by reduction, e.g., via the Trx1 system. If not protected, the oxidation into disulphide bridge formation or higher oxidation states, namely sulfinylation or sulfonylation will take place. To investigate protein sulfenylation, we treated the cells with 10 μM SFN for up to 10 min and monitored protein sulfenylation by dimedone. Dimedone is a cell-permeable reagent that exclusively recognizes and binds to cysteines at their sulfenylated form (28). Figure 4E shows representative N-SIM images of T-cells which revealed a slightly increased global dimedone signal in the cytoplasm and on the cell surface upon SFN treatment. To better quantify the dimedone signal, we, in addition, performed flow cytometric analysis. Similarly, flow cytometry results revealed a significant increase in sulfenylated cysteines after treatment with SFN, as depicted by an increase of the dimedone MFI (Figure 4F). These results imply that SFN increases protein oxidation in primary human T-cells.

### Inhibitory effects of SFN on T-cell activation are abrogated by thiol-containing antioxidants

**Figure 5:**
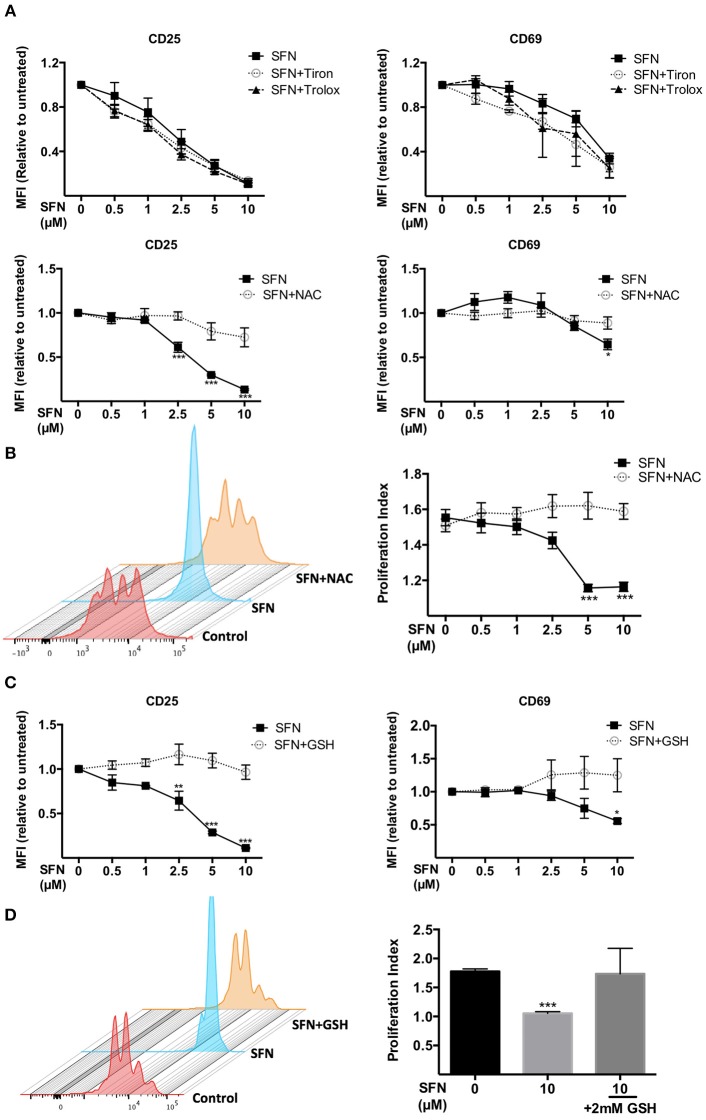
Immunosuppressive effects of SFN were abrogated by thiol-containing antioxidants. **(A,C)** Expression of CD25 (left) and CD69 (right) in T-cells were analyzed by flow cytometry under the indicated conditions. T-cells in the presence/absence of SFN and **(A)** Tiron, Trolox (upper graphs), NAC (lower graphs) or **(C)** GSH were co-stimulated with anti-CD3(20 ng/ml) /CD28(5 μg/ml) antibodies for 1 day. Shown are the MFI ratios of SFN treated to untreated samples (*n* = 3; mean; SE; ^*^p < 0.05, ^**^p < 0.01, ^***^p < 0.001). **(B,D)** T-cell proliferation was detected via staining of CFSE. T-cells were loaded with CFSE, thereafter co-stimulated with anti-CD3 (20 ng/ml)/CD28 (5 μg/ml) antibodies in the absence/presence of SFN without and with addition of exogenous NAC **(B)** or GSH **(D)**. CFSE signals were measured after 3 days of co-stimulation by flow cytometry. Shown are representative histograms (left) and the proliferation index (right) from three independent experiments (*n* = 3; mean; SE; ^***^p < 0.001).

We next aimed to clarify whether the immunosuppressive effect of SFN on T-cells is due to the pro-oxidative capacity of this substance. To this end, we made use of the antioxidants N-acetyl-cysteine (NAC), Tiron and Trolox. Of those only NAC treatment can lead to replenishment of GSH stores (29), while Tiron and Trolox do not. Notably, we observed that only NAC (2 mM), not Tiron or Trolox, reversed the SFN-induced downregulation of CD25 and CD69 (Figure 5A). Moreover, NAC abolished the inhibitory effect of SFN on T-cell proliferation (Figure 5B). This suggested that SFN mainly exerts its inhibitory effects on T-cell functions via oxidation of cysteine residues through depleted GSH stores. To further substantiate this hypothesis, we tested the effects of SFN on T-cell activation in the presence and absence of exogenously added GSH. Indeed, GSH completely prevented the SFN-induced defect in the costimulation-dependent upregulation of CD25 and CD69, and T-cell proliferation (Figures 5C,D). Taken together, these results show that SFN mediates immunosuppressive effects on untransformed human T-cells via depletion of intracellular GSH.

### Gene expression analysis revealed that T_H_17-related genes are highly sensitive to SFN

**Figure 6:**
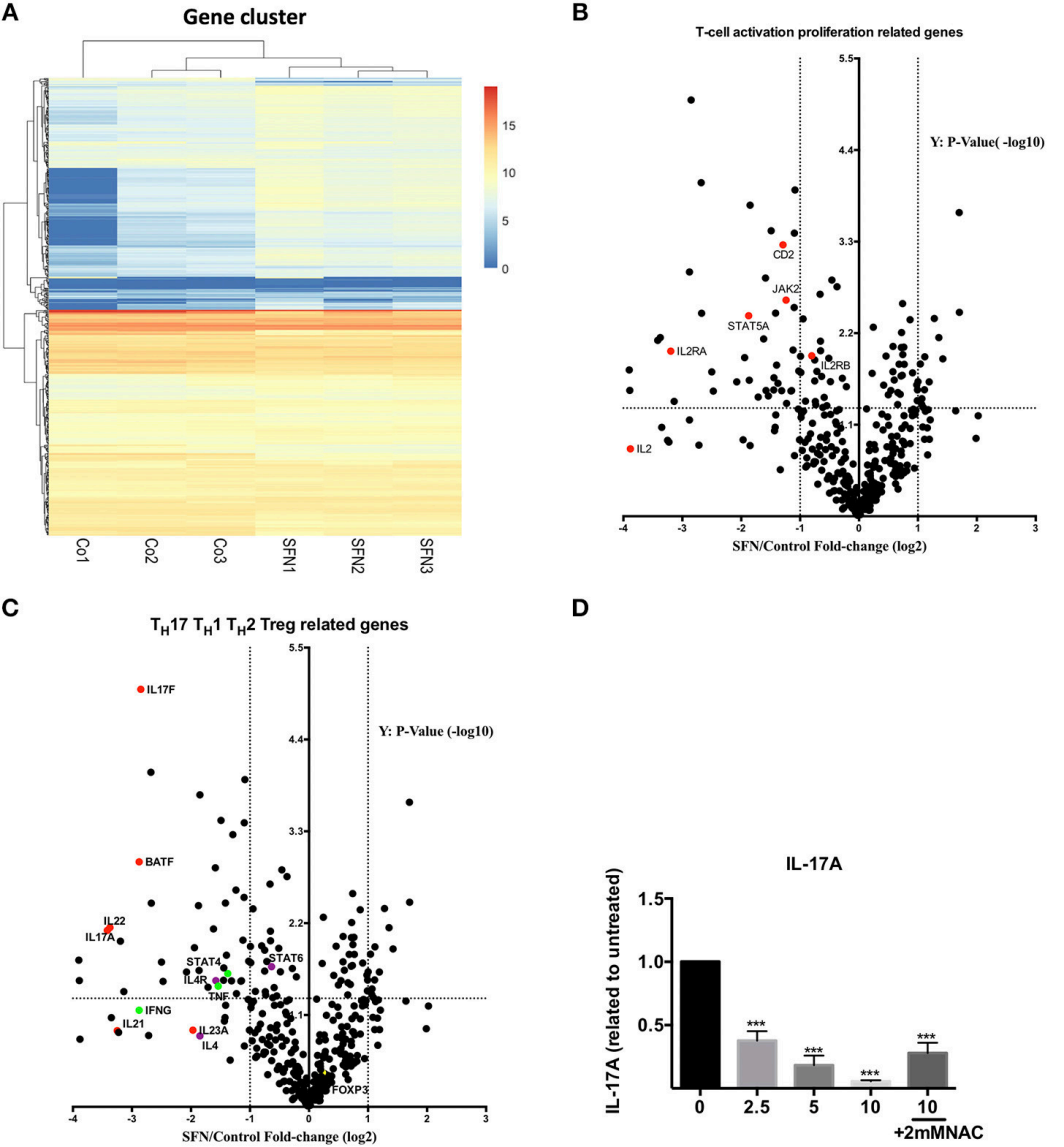
Gene expression analysis (nCounter®). **(A)** Hierarchical clustering of differentially expressed mRNAs in untreated (Co, *n* = 3) and SFN treated (SFN, *n* = 3) T-cells of three different donors. Each column represents an individual sample. Relative fold change is indicated by the color scale (red: high; blue: low). **(B,C)** Volcano plots show the differentially expressed genes important for T-cell activation and proliferation **(B)** and related to T_H_1, T_H_2, Treg, and T_H_17 cells **(C)**. Log2 transformed fold change in expression (SFN vs. control) on the x-axis is plotted against significance (–log10 adjusted *p*-value) on the y-axis. The horizontal dashed lines indicate cutoff for significance *p* < 0.05 (–log10 *p*-value >1.3) and the vertical lines for fold change ≥2/≤-2. **(D)** Quantification of IL-17A in the supernatant of T-cells co-stimulated with anti-CD3 (20 ng/ml)//CD28 (5 μg/ml) antibodies for 2 days in the absence/presence of SFN, with or without NAC supplementary treatment as indicated. IL-17A was measured by a human T_H_ cytokine panel (*n* = 3; mean; SE; ^*^p < 0.05 ^***^p < 0.001).

To get an unbiased view on differential mRNA expression profiles in untransformed T-cells after co-stimulation in the presence vs. absence of SFN, we compared the expression of 594 immune relevant genes using the NanoString GX Human Immunology v2 panel. The evaluated genes were associated with leukocyte functions including major classes of cytokines and their receptors. After normalization of the raw counts based on the six housekeeping genes, datasets from three experiments were visualized by hierarchical clustering (Figure 6A). Upon SFN treatment, we found that 39 genes were downregulated and 13 genes were upregulated (fold change >2, *p* < 0.05) as compared to untreated cells. Notably, consistent with our findings that SFN inhibited CD25 and IL-2 protein expression, the mRNA levels of CD25 (*IL-2RA*) and IL-2 were substantially decreased by SFN treatment (Figure 6B). Moreover, the expression of signal transducer and activator of transcription 5A (*STAT5A)*, an essential mediator of IL-2 signaling in T-cells (30), also decreased with SFN treatment, which may, together with the diminished CD25 expression, explain why the addition of exogenous IL-2 was not sufficient to rescue proliferation of SFN treated T-cells (see above).

Most intriguingly, the expression of T_H_17-related genes such as *IL17A, IL17F, IL22*, and B-cell activating transcription factor (BATF) was strongly decreased by SFN, while T_H_1, T_H_2 or Treg related genes, i.e., *STAT4, IL-4*, and *FOXP3* were no major targets of SFN (Figure 6C). To substantiate the finding that T_H_17-related genes are highly sensitive to SFN-treatment, we measured the amount of IL-17A at the protein level in the supernatants of T-cells pre-incubated in the presence or absence of SFN and costimulated for 2 days. In accordance with the mRNA data, SFN significantly diminished the amount of IL-17A (Figure 6D). Note that supplementary treatment with NAC could rescue the decrease in IL-17A production upon SFN treatment. These data show that SFN strongly affects T_H_17 polarization.

### SFN induces thiol oxidation on STAT3 and inhibits STAT3 phosphorylation

**Figure 7:**
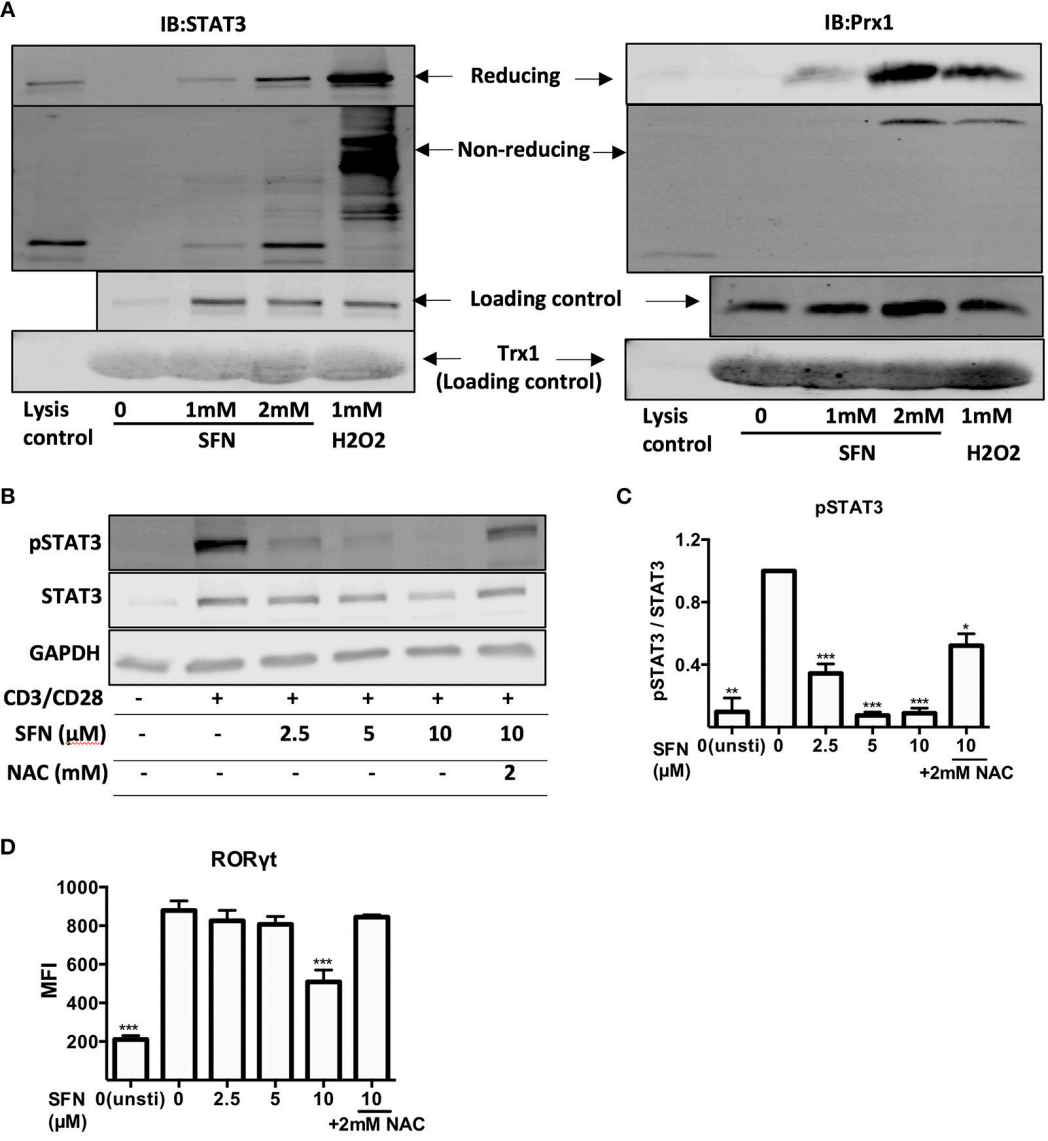
SFN induces thiol oxidation on STAT3 and inhibits STAT3 phosphorylation. **(A)** Oxidized proteins were delineated by Trx1 kinetic trapping. T-cells were treated without or with SFN or H_2_O_2_ as indicated. Cell lysates were incubated with Trx1 trapping mutant loaded streptavidin beads, eluted proteins were concentrated and loaded on SDS-PAGE gel. Shown immunoblots are representative for three experiments. **(B)** Phosphorylation of STAT3 was detected by western blot. T-cells were treated without or with SFN as indicated under stimulated (+) or unstimulated (−) conditions for 1 day. Cell lysate was loaded on SDS-PAGE gel and immunoblotted for pSTAT3, STAT3, and GAPDH. **(C)** Quantification of pSTAT3 to STAT3 (*n* = 3; mean; SE; ^*^p < 0.05, ^**^p < 0.01 ^***^p < 0.001). **(D)** Quantification of RORγt expression by flow cytometry. T-cells were treated with or without SFN as indicated under stimulated or unstimulated conditions for 2 days and then stained with RORγt specific antibody. Given are expression levels relative to control (no SFN treatment) in terms of MFI (*n* = 3; mean; SE; ^***^p < 0.001).

Since T_H_17 related genes were suppressed by SFN on both mRNA and protein level, we next aimed to clarify how SFN affects T_H_17 cells. STAT3 is essential for the differentiation of T_H_17 cells (31, 32). We, therefore, analyzed the regulation of STAT3 at the posttranslational level by means of thiol oxidation on cysteines and phosphorylation upon SFN treatment in primary human T-cells. To this end, a Trx1 kinetic trapping mutant was used to analyze whether SFN induces oxidation of STAT3. Trx1 is an oxidoreductase that resolves oxidized proteins by thiol-disulfide exchange reactions. Mutating the catalytic cysteine (C35S) of Trx1 results in formation of long-lived disulphide bridges with its specific targets, enabling trapping of oxidized Trx substrates (33). Since we intended to trap proteins that were oxidized (before reversal) after a very short time, a bolus of high SFN (mM) or H_2_O_2_ (mM) was chosen to increase the likelihood of oxidized protein detection (34, 35). Using this system, we found that Prx1 [a known target for oxidation that can be trapped by Trx1 (33)] and STAT3 could be trapped with Trx1 following SFN treatment (Figure 7A). The amount of trapped STAT3 as well as Prx1 increased in a dose-dependent manner. This result reveals that SFN leads to oxidation of STAT3 and Prx1 in primary human T-cells.

We next scrutinized whether the SFN-mediated increased ROS/decreased GSH levels in T-cells could affect the costimulation-induced phosphorylation of STAT3. To this end, T-cells were pre-treated with SFN with the indicated concentrations in the presence or absence of 2 mM NAC and were costimulated with crosslinked anti-CD3/CD28 antibodies. The intracellular levels of pSTAT3 were analyzed by flow cytometry (data not shown) or western blot (Figures 7B,C). STAT3 phosphorylation decreased significantly in the presence of SFN. The inhibitory effect was dose-dependent and started already at 2.5 μM SFN. Importantly, this inhibition was significantly reversed by NAC treatment. These data provide strong evidence that oxidative stress/GSH depletion induced by SFN inhibits STAT3 activation in primary human T-cells connecting SFN treatment to T_H_17-skewing of T-cell responses. This notion was further supported by the finding that the expression of RORγt, a master regulator of T_H_17-associated gene transcription (36), was also inhibited by SFN (Figure 7D). Moreover, this inhibition was also reversed by NAC treatment.

### SFN displays an immunosuppressive effect on *ex vivo* T-cells from RA patients and provokes ROS production in whole blood lymphocytes

**Figure 8:**
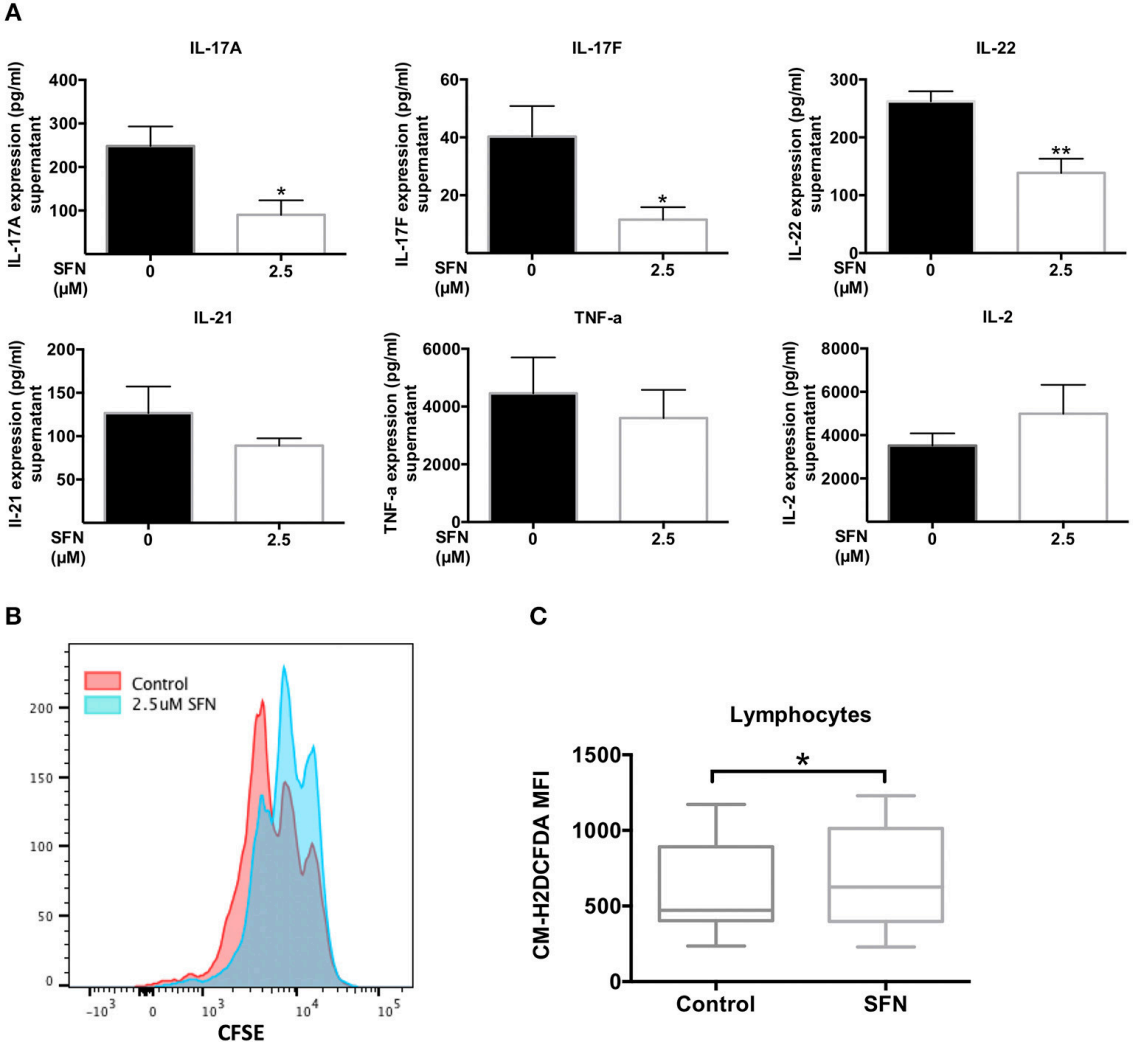
SFN displays an immunosuppressive effect on RA T-cells *ex vivo*. **(A)** Quantification of cytokines in the supernatant of RA T-cells by bead-based immunoassay using the human T_H_ cytokine panel. RA T-cells were purified and co-stimulated with anti-CD3 (20 ng/ml)/CD28 (5 μg/ml) antibodies for 2 days in the absence/presence of SFN. Shown are the cytokine levels in the supernatant in pg/ml (*n* = 4; mean; SE; ^*^p < 0.05, ^**^p < 0.01). **(B)** RA T-cell proliferation was assessed via CFSE staining. RA T-cells were co-stimulated with anti-CD3 (20 ng/ml)/CD28 (20 ng/ml) antibodies in the absence/presence of SFN for 3 days. CFSE signals were detected by flow cytometry. Shown is the representative histogram. **(C)** Intracellular ROS levels of RA blood cells were assessed via staining of CM-H_2_DCFDA. Whole blood was stained with AF700-CD45 and CM-H_2_DCFDA in the absence/presence of SFN. Shown are ROS levels in terms of MFI in both untreated and SFN treated blood lymphocytes (*n* = 17; medium; SE; ^*^p < 0.05).

Low ROS levels in RA patient-derived T-cells were linked to biased differentiation of T-cells into IFN-γ and IL-17-producing inflammatory cells (18). IL-17A, as the major inflammatory mediator (37), increases bone resorption during RA. We have shown here that SFN increased the intracellular ROS levels in untransformed human T-cells and downregulated T_H_17 related genes. Therefore, it was tempting to speculate that an increase of the ROS content in T-cells induced by treatment with SFN should be beneficial for RA patients due to a dampening effect on T_H_17 effector functions. We, therefore, investigated the effect of SFN on purified peripheral blood T-cells from RA patients *ex vivo*. These cells were costimulated for 2 days in the absence or presence of 2.5 μM SFN and cytokines in the supernatant were measured by LEGENDplex™. Figure 8A shows that IL-17A, IL-17F, and IL-22, three important cytokines produced by T_H_17-cells, were significantly decreased when RA T-cells were costimulated in the presence of 2.5 μM SFN. In contrast, TNF-α, IL-2, and IL-21 protein levels were not significantly changed under these conditions. Costimulation-induced proliferation of *ex vivo* RA T-cells was also inhibited by SFN under these conditions (Figure 8B).

Our analyses so far were performed with purified human T-cells. Since there are many buffer systems in the whole blood that could interfere with the effects of SFN observed in purified human T-cells, we next analyzed whole blood samples from RA patients. 10 μM SFN were added to the whole blood samples and the ROS production was analyzed in lymphocytes without a purification step using flow cytometry. These experiments revealed that also in whole blood SFN treatment evoked a significant increase in the intracellular ROS levels in the lymphocyte population (Figure 8C). Taken together, these results show that SFN inhibits the production of T_H_17 related cytokines from RA T-cells and enhances the ROS levels in whole blood lymphocytes of RA patients.

## Discussion

SFN has extensively been studied as an anti-cancer agent, while its impact on untransformed human immune cells remained largely unknown. In our study, the effects of SFN on primary human T-cells were addressed. We demonstrate that SFN does not change immune synapse maturation as determined by clustering of CD3 and the adhesion molecule LFA-1, but it inhibits the activation of untransformed human T-cells, in particular their proliferation and the production of T_H_17-related cytokines. As a major finding, we show that SFN induces a pro-oxidative state in untransformed human peripheral blood T-cells. This manifests as an increased general ROS amount, severely depleted intracellular GSH pools, and oxidation of redox sensitive proteins including the T_H_17-regulating transcription factor STAT3. Seemingly contradictory, various previous studies on tumor cell lines (38, 39) or murine DCs (8, 12) showed that SFN enhanced the cellular antioxidant capacity by increasing KEAP1/NRF2/ARE-dependent expression of phase II antioxidant enzymes, e.g., HO-1. This led to the conviction that SFN generally acts as an antioxidative substance. In this regard, we also observed increased levels of *NRF2* and *HO-1* upon SFN treatment in both untransformed human T-cells (Supplementary Figure 5A) and prostate cancer PC-3 cells (Supplementary Figure 5B). However, the redox regulation within the cells depends on the activity of all ROS producing and eliminating processes (e.g., antioxidant systems) that may vary in different cell types. It is, therefore, crucial to analyze the intracellular net ROS level and its functional consequences, e.g., direct protein oxidation in a time dependent manner.

Mechanistically, depletion of GSH stores in untransformed human T-cells cells was indeed accompanied by increased oxidation of proteins at redox active cysteine residues. Thus, immunostaining against dimedone, a cell permeable cysteine sulfenylation specific reagent, revealed a global increase in oxidation on various proteins. Consistently, our kinetic trapping approach using the Trx1 C35S mutant revealed that well-known redox regulated proteins, such as Prx1, were trapped upon SFN treatment. Interestingly, the thiol antioxidant NAC, which is a precursor for GSH biosynthesis and GSH *per se* could restore T-cell functions in the presence of SFN. Therefore, the SFN effects on T-cell functions can at least partly be explained by depletion of GSH and accumulation of oxidized proteins. In support of this assumption, GSH has been shown to be essential for maintaining T-cell inflammatory responses (40). Note that since GSH binds to several proteins that control cellular processes such as cell proliferation, apoptosis, and survival, it is not only important to mount a proper anti oxidant response, but also important for many cellular functions that are independent of its anti oxidant activity (41, 42). Depletion of GSH by SFN may, thus, further challenge the cells through inhibiting such functions.

To further characterize the effects of SFN on untransformed human T-cells, immune specific gene analysis was used to get an unbiased view on mRNA expression profiles after T-cell co-stimulation in the presence vs. absence of SFN. Intriguingly, it revealed that SFN especially dampened the expression of the T_H_17-related genes *IL17A, IL17F, IL22*, and *BATF*. Importantly, using a Trx1-trapping mutant we found that the T_H_17-skewing protein STAT3 is oxidized upon treatment with SFN. It was previously shown that ROS can lead to oxidation of STAT3 (43), but the effect of oxidative stress on STAT3 signaling was reported divergently. On the one hand, ROS in higher concentration, are likely to influence STAT3 signaling indirectly by inhibiting protein tyrosine phosphatases and activating protein kinases, which in turn increases STAT3 phosphorylation (44, 45). On the other hand, Halvorsen et al. reported that oxidative stress blocks tyrosine phosphorylation of STAT3 and the activation of JAK/STAT signaling in neurons (46, 47). According to this, the STAT3 phosphorylation and the STAT3 oxidation state are likely to be dependent on cell type and context. As revealed in our study, in untransformed human T-cells SFN led to STAT3 oxidation and inhibited STAT3 phosphorylation. Notably, this inhibition could be rescued by NAC. Fu et al. have demonstrated that ROS activated Mink1 inhibits phosphorylation of T324 in Smad2 and its nuclear localization, thereby preventing the induction of T_H_17-associated genes (48), e.g., STAT3. Whether this pathway is involved in SFN induced dephosphorylation of STAT3 remains to be elucidated, but the ROS-mediated regulation of STAT3 may partially connect SFN-treatment to the strongly inhibited T_H_17 cell responses on the molecular level. Note that Chaudhry et al. have shown that the activation of STAT3 in Tregs endows them with the ability to suppress T_H_17 responses through increasing the expression of a subset of suppressor molecules (49). Therefore, STAT3 activation in Treg vs. T_H_17 cells seems to lead to a different outcome regarding the net activation of T_H_17 cells. In our study, the inhibited STAT3 activity seems to play more a role in T_H_17 cells, since the T_H_17 cell response was clearly inhibited. Yet, to clarify the specificity of the effects of SFN on STAT3 in different cell populations, further experiments need to be performed in the future.

T_H_17 cells have been involved in the progression of many common autoimmune diseases, including psoriasis (50), inflammatory bowel disease (IBD) (51), multiple sclerosis (MS) (52), and rheumatoid arthritis (RA) (53). Although IL-17, as a hallmark cytokine of T_H_17 cells, plays critical roles in the pathogenesis of autoimmune diseases, targeting IL-17 alone with anti-IL-17 antibodies was not sufficient to improve clinical end points (54). Recently, the regulatory effects of ROS in autoimmune inflammation have received more and more attention. While the role of ROS in the pathogenesis of psoriasis is still controversially discussed (55–57), it plays a major role in the pathogenesis of IBD and MS. Moreover, Weyand et al. recently reported that RA T-cells are distinguished from healthy T-cells based on diminished ROS production thereby undergoing “reductive stress” (18). It shunts glucose away from pyruvate and ATP production toward the pentose phosphate pathway, where NADPH is generated and cellular ROS are consumed (58). These ROS^low^ RA T-cells are spontaneously biased to develop into IFN-γ and IL-17 producing pro-inflammatory T-cells, which play a central role for disease progression (58, 59). Therefore, strategies that are able to upregulate ROS concentrations in RA T-cells and to re-balance the ROS signaling systems may be promising for therapeutic purposes. In light of these findings, we extended our studies to T-cells derived from RA patients. *Ex vivo* experiments showed that SFN indeed enhanced the ROS levels in lymphocytes within whole blood of RA patients and inhibited production of the pro-inflammatory cytokines IL-17A, IL-17F, and IL-22. These results suggest that SFN may act as a promising substance to control RA in patients. Supporting this assumption, it was demonstrated in mice that SFN dampened the clinical severity of experimental arthritis by inhibiting the production of cartilage destructive metalloproteinases and the expression of IL-17 and TNF-α (9, 60).

Also, of high clinical importance may be our second finding, namely that SFN has differential effects on untransformed human T-cells (decrease of GSH and immunosuppression) and Jurkat T-leukemia cells—prototypical immature transformed T-cells (increase of GSH and cytotoxicity). This finding may be explained by the different anti oxidant capacity of tumor cells vs. untransformed cells. Tumor cells are usually equipped with stronger anti oxidant systems (e.g., GSH and Trx1 systems) and can keep their redox balance more stable (61). For example, the cystine/glutamate antiporter SLC7A11, the main route of cysteine acquisition, and the glutamate cysteine ligase modifier subunit (GCLM), which is necessary for the efficient synthesis of GSH, are upregulated in the tumor cells during oxidant stress to increase the GSH synthesis. Aside from the biosynthesis, tumor cells can regenerate GSH by upregulating the production of NADPH as well. Collectively, ROS induction in tumor cells leads to a positive feedback on their antioxidant capacities. It is, therefore, also expected to observe an increased GSH in Jurkat T-leukemia cells over time with SFN treatment (62). Not only death of malignant cells but also a functional immune response is crucial for tumor rejection. Therefore, our finding that SFN has an immunosuppressive effect on untransformed human T-cells may explain why treatment with SFN did not lead to amelioration of cancer severity in patients as demonstrated in a number of clinical trials (4), although *in vitro* experiments on tumor cells such as breast cancer cell and hepatocellular carcinoma cell strongly suggested that SFN has anti-tumor properties (2, 63).

Together, in our current study, we uncovered a so far unknown molecular mechanism of how SFN controls activation of human T-cells and, notably, its strong effects on T_H_17-related genes: SFN is able to create a pro-oxidative ROS enriched milieu in primary human T-cells. It inhibits costimulation-initiated T-cell activation and proliferation by depletion of GSH and oxidation of proteins at redox active cysteine residues. Importantly, SFN also enhanced the ROS levels in lymphocytes within whole blood of RA patients and inhibited the production of pro-inflammatory T_H_17 related cytokines. This suggests that SFN may offer a new therapeutic option for the treatment of chronic T_H_17 related diseases, e.g., rheumatoid arthritis, due to its redox-related immunosuppressive effects. At the same time, it may be potentially harmful in cancer settings, in which the T-cell mediated defense of tumors plays a decisive role.

## Author contributions

JL and YS: conceptualization; JL, BJ, EB, GW, and BN: methodology; JL, BJ, JZ, and EB: investigation; YS and NB: resources; JL, EB, GW, KH, and YS: writing; YS: supervision; YS: funding acquisition; All authors have given approval to the final version of the manuscript.

### Conflict of interest statement

The authors declare that the research was conducted in the absence of any commercial or financial relationships that could be construed as a potential conflict of interest.
