# Supplementary material for: Sulforaphane Inhibits Inflammatory Responses of Primary Human T-Cells by Increasing ROS and Depleting Glutathione
Source: Front Immunol. 2018 Nov 14;9:2584. doi: 10.3389/fimmu.2018.02584 (PMC6246742; doi:10.3389/fimmu.2018.02584)
Supplement: Supplementary file 1 [file Data_Sheet_1.docx]

Supplementary Material

**Sulforaphane Inhibits Inflammatory Responses of Primary Human T-cells by increasing ROS and depleting Glutathione**

Jie Liang^1^, Beate Jahraus^1^, Emre Balta^1^, Jacqueline D. Ziegler^1^, Katrin Hübner^1^, Norbert Blank^2^, Beate Niesler^3, 4^, Guido H. Wabnitz^1^, and Yvonne Samstag^1,^ *

***Correspondence:** Prof.Yvonne Samstag: [yvonne.samstag@urz.uni-heidelberg.de](https://wwwmail.urz.uni-heidelberg.de/horde/imp/message.php?index=2592)

# Supplementary Data

## Mitochondrial Membrane Potential analysis

The Mitochondrial Membrane Potential detection kit (MitoPT TMRM) was purchased from ImmunoChemistry Technologies. For measurements of mitochondrial membrane potential, tetramethylrhodamine, methyl ester (TMRM) was added at a final concentration of 40 nM to SFN treated or untreated cells and incubated for 15 min at 37 °C. Fluorescence intensity was determined by flow cytometry (LSRII, BD Bioscience, Heidelberg, Germany) immediately after staining. All data were analysed with FlowJo X (FlowJo LLC, Ashland, OR, USA).

# Supplementary Figures

## Supplementary Figure 1

**Supplementary Figure 1.** **SFN does not influence the mitochondrial membrane potential of T-cells.** Mitochondrial membrane depolarization in T-cells was analysed by flow cytometry using the MitoPT TMRM. T-cells were treated without or with SFN for 1 day as indicated, carbonyl cyanide 3-chlorophenylhydrazone (CCCP) was used to create a positive control cell population containing depolarized mitochondria. T-cells were then stained with MitoPT TMRM and measured by flow cytometry (n=3; mean; SE; ****p<0.001*).

## Supplementary Figure 2

**** ****

**Supplementary Figure 2.** **Decrease of total CD25 and CD69 expression by SFN**. Flow cytometry analysis of total expression of CD25 and CD69 in T-cells. After SFN treatment and co-stimulation with anti-CD3(20 ng/ml)/CD28(5 µg/ml) antibodies, T-cells were fixed, permeabilized and stained for CD25 and CD69. Given are expression levels relative to control (CD3/CD28 co-stimulation, no SFN treatment) in terms of %positive cells and MFI (n=3; mean; SE; **p<0.05*, ***p<0.01*).

## Supplementary Figure 3

**Supplementary Figure 3. Exogenous IL-2 treatment cannot reverse T-cell activation**. Flow cytometry analysis of CD25 and CD69 surface expression in T-cells. T-cells in the presence/absence of SFN were treated with 40 U IL-2 or not. Given are MFI ratios of SFN treated samples to untreated samples (n=3; mean; SE; ***p<0.01*, ****p<0.001*).

## Supplementary Figure 4.

**Supplementary Figure 4. SFN treatment induces opposite effects on GSH levels in T-cells and Jurkat T-leukemia cells.** Intracellular GSH levels were assessed via staining with GSH specific thiol green dye. T-cells and Jurkat T-leukemia cells were treated with SFN as indicated for 1 d and stained with thiol green dye. The GSH signal was detected by flow cytometry (n=3; mean; SE; **p<0.05*, ***p<0.01*).

## Supplementary Figure 5.

**B.**

**A.**

**Supplementary Figure 5. SFN treatment increases expression of antioxidant genes**. Gene expression analysis of mRNA levels of NRF2 and HO-1 in T-cells **(A)** and PC-3 cells **(B)**. Cells were treated with SFN, thereafter, total RNA was extracted and detected by the nCounter® analysis system (n=3).
